# Women as a driver to address gaps in the global surgical workforce

**DOI:** 10.1186/s12960-023-00808-6

**Published:** 2023-03-16

**Authors:** Isabella Busa, Shobhana Nagraj

**Affiliations:** 1 University of Oxford Medical School, Osler House, John Radcliffe Hospital, Oxford, UK; 2 Oxford University Global Surgery Group, Nuffield Department of Surgical Sciences, University of Oxford, Oxford, UK; 3 Health Systems Collaborative, Oxford Centre for Global Health Research, Nuffield Department of Medicine, University of Oxford, Oxford, UK

**Keywords:** Global surgery, Women in surgery, Surgical workforce, LMIC

## Abstract

Five billion people around the world lack access to safe and affordable surgical, anaesthetic, and obstetric care. There is a link between countries in which women are underrepresented in the surgical workforce and those struggling to meet their surgical need. In this commentary article, the underrepresentation of women in low- and middle-income country’s (LMIC) surgical workforce is discussed. It is argued that the issue is self-reinforcing. On one hand, active change requires a sufficient number of female surgeons to initiate it. On the other, women can only start to penetrate the surgical workforce once they are safe, healthy, and motivated enough to do so, in turn depending on the presence of female surgeons to advocate for their female patients and empower future generations of young girls and women.

## Introduction

In 2015, the Lancet Commission estimated that 143 million additional surgical procedures were required each year to address the unmet surgical need in LMICs [1], a number that has continued to grow. Globally, approximately one-third of all disease are due to surgically treatable conditions; this burden is greater than that of HIV/AIDS, malaria, and TB combined [1]. It is reported that there are only 3 female surgeons for every 1 million people in LMICs [2]. Although the surgical capabilities of these countries can be improved through investment in medical equipment and surgical training programs, expanding participation of women in surgery remains a large, untapped resource to alleviate the global surgical burden.

## Barriers for women pursuing a surgical career

The barriers for women pursuing a surgical career, both perceived and systemic, transcend diverse political, cultural, and economic contexts, thereby limiting not only the number of female surgeons, but also the number of women in surgical leadership positions [3, 4]. Such barriers include factors both preventing women from choosing a surgical career and those promoting their drop-out, such as negative lifestyle perceptions, a lack of mentors and female role models, persistent and pervasive ‘old boys club’ attitudes, and unequal pay [5, 6].

Implicit and explicit gender biases are manifest in the day-to-day work environments of female surgeons: they are less often introduced by their title, are commonly mistaken for non-physician members of their team [5], and are less able to gain patients’ trust and confidence due to public preconceptions of surgeons generally as male [5]. Women also report motherhood penalties, whereby career opportunities are constrained irrespective of personal desire or ability to have children, due to the female sex being equated with a mandate for motherhood [5].

It is true that several challenges face both men and women pursuing careers in surgery, such as long working hours and unavailability of leave, and the impact of such professional expectations on personal relationships. However, even these general challenges affect women disproportionately. Persistent societal expectations of a gendered division in domestic responsibilities mean women in high-achieving cohorts still spend on average 8.5 h more per week on parenting and domestic tasks than men [7]. Furthermore, only women face the physical consequences of pregnancy, parturition, and lactation; the peak years of fertility coinciding with those of postgraduate surgical training.

In addition to these pervasive challenges, women in LMICs face further specific barriers to pursuing surgical careers. Traditional cultural attitudes to female education remain and may foster discouragement and disapproval by family or close friends [8]. Women in both high- and low-income countries are caught between societal norms and their career ambition, where implicit biases dominate in the former, explicit and widely accepted cultural barriers, especially towards education, are more important in the latter.

While all these challenges accumulate to disadvantage women in surgery, none will change passively. To wait patiently for gender biases to disappear is a frequently proposed solution, since more and more women continue to choose careers in surgery. However, to accept the continued attrition and underrepresentation means we will necessarily tolerate lower wages, stunted career progression, and reduced work satisfaction for women in the meantime. Instead, policy change must be aligned to areas, where female surgeons can actively lead and affect targeted change.

Simply educating surgeons themselves is not enough. One study examining the gender-based discrimination reported by female trainees in surgery and allied careers found that, although surgeons were by far the greatest perpetrators, a substantial proportion of discrimination experienced was by nurses [9]. This indicates that gendered beliefs transcend the surgical team and reinforces a need for systemic reform that encompasses all aspects of a female surgeon’s working environment and wider public perception.

## The argument for including women in the surgical workforce

Besides representing an important untapped source of potential surgical workforce, the inclusion of women in surgical teams has other important benefits. First, performance statistics are consistently superior: patient outcomes, including hospital mortality and readmission rates, are improved when treated by female physicians and female surgeons [10]. Women perform comparably to, if not better than, their male peers when measured on medical knowledge, clinical judgment, technical and communication skills, and professionalism [6].

Second, female surgeons are poised to be powerful advocates for female patients and for fellow female colleagues. This mirrors the case for racial and ethnic diversity in healthcare workforces to deliver culturally competent care and assure awareness of issues unique to minority populations. Finally, the surgical environment is more welcoming and cooperative when women are included [11] and sexual harassment is more common in environments, where women fail to share equal power [12]. A key component of improving medical workplace culture more generally, then, must involve the acceptance, inclusion, and promotion of women to the ‘room where it happens’.

Some argue that narrowly targeted, women-only interventions can result in adverse consequences, such as reinforced stereotypes and a female-deficit approach [13]. Indeed, there is a growing body of international experience that suggests gender equality is accelerated most when a systems or whole-institution approach is taken. Thoughtful and considered changes can clear the way for both men and women but are likely to disproportionately benefit women by virtue of the increased number of obstacles they face. Likewise, interventions targeted at all those facing demands of extra-professional caregiving will disproportionately benefit women, since they are more likely to serve in such domestic roles [14]. Similarly, the cultivation of a respectful and inclusive work environment is essential for productivity but will be of particular importance for women, who are currently most discriminated against.

## Many proposed solutions are not applicable to LMICS

Despite such numerous and clear benefits, and an increasing awareness of the issue in the literature and wider consciousness, the number of female surgeons in LMICs is rising more slowly than female representation in other medical specialties [4]. This is due, in part, to the continued pursual of a ‘one-size-fits-all’ fix. Rather, the diversity of challenges facing women in surgery calls for similarly varied solutions. Many of the commonly proposed solutions to gender inequalities in the surgical workforce, such as promoting equity in training (women receive less formative feedback and less theatre autonomy than their male peers [5]), offering paid maternity leave, and challenging unconscious gender biases by promoting the use of formal titles and challenging regressive attitudes, do not target the fundamental root of the issue in LMICs.

For instance, mentoring is particularly promoted to enhance the female leadership pipeline and marshal the empowerment of women in surgery. However, due to the lack of female representation, and access to training opportunities, women are often not in positions from which they can be successfully mentored into surgical leadership positions. In the context of stark inequity in educational opportunity, how, then, will creating more training positions and mentors to guide professionally qualified women into surgical specialities improve the more systemic issues affecting female education?

## Female under-representation in the LMIC surgical workforce is self-reinforcing

Crucially, the lack of women in LMIC surgical workforces is self-reinforcing. Since women have the ability to empower women, as is the premise of much of the HIC-centred discussion around surgical gender equality, women are required as advocates and figureheads to push through change and break many of the vicious cycles, such as experiences of violence and poorer health outcomes, which continue to limit the life-expectancy, and thereby professional development, of women in LMICs [15].

It is because of the positive impact of women in surgery that the overall growth and success of women in LMICs will continue to be stifled until women are included in medical workforces. Women improve health outcomes for female patients [16]; this is in turn fundamental to their ability to attend education, achieve qualifications, and pursue high-achieving careers. For instance, cervical cancer disproportionately affects poor women [17] and LMICs account for 90% of deaths worldwide [18]. The increased presence of women in medical spaces will help shift focus onto such neglected, often exclusively female, conditions.

Female doctors in the community may help reduce violence against women and girls, such as by providing crucial trauma and gynaecological care recommended to those experiencing female genital mutilation [19]. Female medical personnel may also increase access to surgical treatment, such that preventable maternal morbidity and mortality can be addressed.

Women may also advocate for and empower women to make decisions about sexual and reproductive health, including access to safe abortion and permanent sterilisation. The implications of this point alone on the educational attainment and professional development of women must not be understated. The presence of women in positions of professional and academic excellence improves visibility, fostering interest and ambition for women and girls in these career pathways; this is arguably the very root of the issue in LMICs.

## Strengths and weaknesses

The thesis of this article—that female underrepresentation in the LMIC surgical workforce is self-reinforcing—is supported by the cited literature. Yet, it is true that some positive change is already afoot and that the landscape may not be as bleak as that portrayed here. For instance, the College of Surgeons of East, Central, and Southern Africa (COSESCA) have introduced a scholarship program to support women in surgical residency programmes to complete their training and encourage other women in medicine to consider surgery as a career [20]. The aligned ‘Women in Surgery Africa’ (WiSA) movement has further promoted female mentorship within the field [21]. Initiatives such as these have already increased the number of women in surgical training pathways [22]. In December 2022, COSECSA elected its first female President [23]. Inclusion of women in positions of leadership and within surgical training pathways can help inform and advocate for their specific needs within the LMIC medical workforce. These steps may, in time, influence the future career choices of female medical graduates, which have significant gendered influences in relation to surgery [24]. Representation of women in professions allied to surgery, such as surgical nursing, has not been systematically reviewed here, largely due to the lack of literature currently available. Nonetheless, this remains an important direction for future research, in order that female representation in positions of leadership and seniority in surgery can be comprehensively reviewed.

## Conclusions

Each of these factors are self-reinforcing: only once women are safe, healthy, and driven to achieve their goals will they start to penetrate the surgical workforce and advocate for further change. Active change will only be achieved once there are sufficient women in these positions to initiate it. It is hard, therefore, to identify where to break the cycle.

In order that any progress is made, it is important to acknowledge that smaller steps can contribute to a larger solution, whereby change is enacted at the level of all stakeholders—regulatory bodies, professional associations, educational institutions, surgical communities, and the colleagues of all genders that comprise them. Considering potential initiatives at each level of this ladder is a good place to start; scholarships represent one such intervention enacting progress at the level of educational institutions which thereby increases the number of women in surgical communities. The development of local champions and role models for women in surgery may also break down some of the socio-cultural barriers facing women pursuing a surgical career.

By raising awareness in articles such as this, one hopes that professional associations and regulatory bodies might soon prioritise the issue and increase the representation and status of women within their organisations. The argument presented here, that increasing female representation represents a viable strategy by which to address the surgical disparity that currently threatens LMIC healthcare, represents an important incentive to enact progress more systemically.

## Data Availability

Not applicable.
